# Does attitude importance moderate the effects of person-first language? A registered report

**DOI:** 10.1371/journal.pone.0332733

**Published:** 2025-10-08

**Authors:** Sandy Schumann, Hazem Zohny

**Affiliations:** 1 Department of Security and Crime Science, University College London, London, United Kingdom; 2 Oxford Uehiro Centre for Practical Ethics, University of Oxford, Oxford United Kingdom; Yeditepe University, TÜRKIYE

## Abstract

Previous research has demonstrated that exposure to outgroup descriptions that use person-first, as compared to identity-first, language can attenuate negative stereotypes or prejudice and enhance support for policies that seek to advance outgroup rights. However, the benefits of person-first language may not apply to all social groups equally. Specifically, we postulate that person-first language reduces the stigmatization of outgroups, but to a lesser degree if individuals hold more important negative attitudes towards the respective communities. We tested this hypothesis in a two-factorial 2 (target group) x 2 (descriptor) online experiment that included a control group and for which we recruited a general-population sample (*N* = 522). Stereotyping, dehumanization, as well as negative affect and approach intentions towards two outgroups were compared: people with a physical disability (i.e., negative attitudes were expected to be less important) and people who have committed a violent crime (i.e., negative attitudes were expected to be more important). Results showed that attitudes towards violent crime were more important than those towards physical disability; other than predicted, attitudes towards physical disability were strongly positive. Importantly, this registered report confirmed that the impact of exposure to person-first language cannot be generalized across social groups. Contesting previous work, exposure to the descriptor “people with a physical disability” (rather than “the physically disabled”) reduced positive stereotypes and increased dehumanization; there was no impact on negative stereotypes, affect, or outgroup approach intentions. Exposure to the descriptor “people who have committed a violent crime” (rather than “violent criminals”), in turn, did not impact positive stereotypes and dehumanization, but negative affect and stereotypes were reduced, and the willingness to approach the group was increased. Exploratory analyses did not provide convincing evidence that attitude importance modulates the size of the effect of person-first language. Future research ought to consider alternative moderators, such as the perceived mutability of stigmatized attributes.

## Introduction

In recent years, several professional organizations have released guidelines that encourage the adoption of inclusive, non-stigmatizing language [1–4]. Noteworthy is the recommendation to use person-first (or person-centered) instead of identity-first language, which is promoted especially in medical and criminal justice contexts [5]. In practice, when communicating with or about an individual, it is, for example, suggested to say “person with autism” and not “autistic”. Or, rather than “ex-convict”, the phrase “person who was formerly incarcerated” is preferred (i.e., a post-modified noun that refers to a person is followed by a descriptor).

Despite criticism of person-first language policies [6–8], empirical evidence points to their benefits. More precisely, exposure to outgroup descriptions that employ person-first language has been found to reduce negative outgroup stereotypes, prejudice, and stigma [9–12]. Having said this, it is also evident that person-first language may not affect perceptions of and attitudes or behavior towards all social groups equally strongly. St. Louis [13] showed that across 12 different descriptor pairings, person-first language evoked more positive impressions for only two outgroups - “persons with leprosy” and “persons with psychosis”. Additionally, effect sizes that were attained in studies that explored the influence of person-first language in medical and criminal justice contexts varied widely [11,14]. The reasons for those differential result patterns were not examined systematically.

The present research addresses this gap in the literature. We investigated whether attitude importance moderates the effects of person-first language such that the latter are weaker for outgroups towards which individuals hold, at the outset, more important *negative* attitudes [15,16]. Testing this hypothesis, we recruited a general-population sample and conducted a two-factorial online experiment to compare negative and positive stereotyping, dehumanization, negative affect, and approach intentions directed at others who have committed a violent crime/violent criminals (i.e., negative attitudes were expected to be more important) and people with a physical disability/the physically disabled (i.e., negative attitudes were expected to be less important).

### Person-first language and its benefits

Efforts to promote person-first language [1–4], which have their origins in the disability rights movement in the 1970s, the 1980s Denver Principles, as well as prison reform and Black liberation movements in the 1960s-70s [17], aim to alleviate stigmatization and its detrimental implications for people's mental/physical health, access to support, and social inclusion [17,18]. Referring, for instance, to someone as “a person with an addiction” (rather than “an addict”) is thought to emphasize their personhood; “humanity (and dignity is preserved) while promoting … individuality” [7], p. 258]. Moreover, person-first language is expected to ensure that addictive behavior, in this example, is viewed as just one of many aspects that shape someone's identity [5,19]. In avoiding essentialization, it is also indicated that the stigmatized characteristic or behavior can be changed [6].

To date, empirical evidence that confirms the benefits of person-first language is limited to studies that have examined its influence on outgroup perceptions, attitudes, and behavioral intentions [20–23] (see Lynch and Groombridge [24] for nil effects and Feldman et al. [25] who demonstrated no relationship between the *use* of person-first language and attitudes towards individuals with disabilities). Ashford and colleagues [9] employed the Go/No-Go Association Task to compare implicit negative biases towards individuals described by the terms “substance abuser” or “person with substance use disorder”. The authors found that significantly more negative implicit outgroup attitudes were associated with the label “substance abuser”. Endorsing these findings, “addicts” were viewed as more inferior than “people with addiction”, and participants endorsed maintaining social distance to “addicts” more strongly; sympathy and helping intentions were also lower for “addicts” [10]. Furthermore, a study that recruited professional counselors reported that participants expressed higher levels of tolerance towards “people with mental illnesses” than “the mentally ill” [14].

Beyond the medical domain, the effects of person-first language have been documented in two studies pertaining to the criminal justice context [18,26]. Reading instructions that referred to another individual as an “offender” elicited more stigma and negative judgments than instructions that made reference to a “person with a conviction” [11]. Describing individuals either as a “returning citizen”, “a person who was formerly incarcerated” (i.e., two different person-first descriptors), or an “ex-convict” (i.e., identity-first descriptor) also affected outgroup perceptions and intentions to support reintegration efforts [12]. Notably, negative stereotypes were increased, willingness for social closeness was reduced, and reintegration measures were less strongly endorsed when participants were exposed to an identity-first descriptor [12].

### Criticism of person-first language

Despite those promising findings, person-first language has also attracted criticism. First, identity-first, not person-first, language is thought to enable a higher expression of agency and autonomy, allowing individuals and communities with lived experience to claim terms like “autistic” or “disabled” [20]. Moreover, it has been argued that separating a person from, for instance, their smoking behavior could suggest that the individual is otherwise not valuable [6]. Additionally, person-first language may highlight that certain behaviors or qualities are negative, thus promoting stigmatization [20,21]. Speaking to this point, Gernsbacher [8] showed that person-first language was “more frequently (used) to refer to children with disabilities than to refer to children without disabilities” (para. 8), and it was, overall, more frequently used to denote more stigmatized characteristics.

Relatedly, person-first language might not always align with someone's self-image [6] or how people prefer to describe themselves. A study with participants who had received an autism diagnosis demonstrated that there were no differences in the preference for the terms “autistic”, “person on the autism spectrum”, or “autistic person” [27]. However, the aforementioned three terms were more preferred than “person with autism”, “person with autism spectrum disorder”, and “person with autism spectrum condition”. Thus, person-first language was evaluated less positively than identify-first language if it specified a disorder or condition [27,28].

### Generalizing the person-first language effect

A further concern regarding the use of person-first language that has not been explored systematically pertains to the generalizability of its benefits. In reviewing the literature, it is evident that person-first language does not affect outgroup attitudes and behavioral intentions to the same degree across different social groups. For example, the prevalence of negative implicit associations differed to a moderate extent between participants exposed to the terms “substance abuser” or “person with a substance use disorder” (d = .39; [9]). A moderate-large difference in levels of sympathy or willingness to help was documented when others were described as “addicts” instead of “people with addiction” (d = .61; [10]; see Granello and Gibbs [14] for moderate-large effects when comparing attitudes towards “people with mental illnesses” and “the mentally ill”). Exposure to person-first rather than crime-first language, in turn, led to only a small decrease in the perceived recidivism risk of “violent offenders” (d = .21; [11]). The same study showed that person-first language did not impact perceptions of those who had committed nonviolent property or drug offenses ([11]; see Jackl [12] for further small effects comparing person- and crime-first language).

Similarly, St. Louis [13] demonstrated that person-first language evoked more positive outgroup impressions for only two of twelve descriptor pairings– “person with leprosy”/”leper” and “person with psychosis”/”psychotic”. These findings were attained in a diverse sample that included individuals with a language disorder, parents of clients, speech-language pathology students, and the general public. St. Louis [13] did not elaborate on why person-first language effects were not identified for all descriptor pairings.

### Attitude importance as a moderator of the effects of person-first language

Advancing the literature, we seek to investigate one potential explanation for the differential result patterns presented in the previous section. As noted, person-first language is expected to attenuate stigmatizing views (and behavior) such that more humanizing outgroup attitudes (and actions) are adopted. However, exposure to outgroup descriptions that use person-first language should only influence attitudes, or influence those attitudes more substantially, that are, in fact, malleable. The latter is not the case for all viewpoints, and there is evidence to suggest that attitudes that are *stronger* are more resistant to change [29–33].

Attitude strength is distinguished from valence. Even if two persons evaluate an object negatively (i.e., same valence), the strength of the respective negative attitudes can vary [34]. Attitude strength also varies between objects, and someone might hold stronger or weaker attitudes about different objects [16]. Furthermore, groups of people (i.e., issue publics [35]) may be characterized by equally strong attitudes towards the same object [36]. That is, attitude strength can fluctuate collectively with the political climate and public attention on certain issues.

A common operationalization of attitude strength is attitude importance, “the subjective sense of concern, caring, and the significance that an individual attaches to an attitude (towards a given object)” ([37], p. 209). Boninger and colleagues [38] stipulated that attitude importance reflects a belief about the significance of an attitude for oneself and forms part of one's self-concept [39]. Thus, this subjective judgment is thought to be independent of notions of descriptive or prescriptive importance (i.e., what attitudes most people attribute significance to or what is the right attitude to attribute significance to).

Three predictors of attitude importance have been identified [15]. An attitude is typically more important if the object it pertains to is of higher material self-interest (i.e., impacts the individual's rights, privileges, or lifestyle), if the attitude (object) is of higher importance to a social group that the individual strongly identifies with, and if the attitude (object) reflects or makes reference to core moral, ideological, or aesthetic values [38]. Self-interest must be differentiated from being personally affected by a situation or condition (i.e., personal relevance/involvement) [15]. In Boninger and colleagues’ [38] seminal work it was also demonstrated that self-interest, social identification, and values influenced the importance of distinct attitudes to different degrees. On the issue of racial segregation, for example, self-interest primarily informed attitude importance; regarding the topic of abortion, self-interest and values predicted the degree of importance at comparable levels [38].

A burgeoning body of research has shown that more important attitudes are more resistant to change even if the person is confronted with substantial contesting evidence [40]. For example, in a field study that examined the impact of participating in a women’s studies class on egalitarian attitudes, the latter were less affected if students viewed the class as more important [41]. In turn, inoculation (or forewarning) messages did not foster further resistance (i.e., predicted less attitude change) for individuals who considered their attitudes on the topic more important; forewarning did boost resistance for those who attributed low significance to the issue [42].

The persistence of more important attitudes has been explained by focusing on cognitive and affective processes. The elaboration likelihood model of attitude strength [43] posits that information associated with important attitudes is processed in a more elaborate manner, which would enhance attitude accessibility and certainty, as well as knowledge about the attitude object. Indeed, important attitudes are typically more salient [32,43]. Additionally, if an important attitude was contested, for instance, by counter-attitudinal information, more negative affective responses such as anger and irritation were evoked (counter-arguing/negative thoughts were also enhanced and the source was viewed less favorable) [39]. These affective responses partially explained the stability of more important attitudes [37,39]. In other words, in line with cognitive dissonance theory [44], individuals experienced more discomfort after encountering information inconsistent with more important attitudes, that is, a negative affect that they seek to avoid.

### The present research

This registered report integrates evidence on the impact of person-first language with these insights about the malleability of (more) important attitudes. First, aiming to replicate previous studies, we postulate that one-time exposure to outgroup descriptions that use person-first language reduces negative outgroup stereotypes (*Hypothesis 1a*) and negative outgroup affect (*Hypothesis 1b*), and increases outgroup approach intentions (*Hypothesis 1c*). We also consider further outcomes, which reflect several unexplored potential benefits of person-first language [5,19]. More precisely, we propose that one-time exposure to outgroup descriptions that employ person-first language fosters stronger positive stereotypes (*Hypothesis 1d)* as well as lower levels of dehumanization of the target outgroup (*Hypothesis 1e*).

Second, we test a boundary condition of the influence of person-first language to investigate why its effects on outgroup perceptions or behavioral intentions vary in size across different social groups—especially when comparing research that was conducted in the medical and criminal justice context. As discussed, it is conceivable that individuals attribute different levels of importance to their attitudes towards various outgroups. Attitudes towards an outgroup are expected to be more important if the outgroup’s actions impact the individual’s rights or lifestyle more strongly (i.e., self-interest); if the attitude about the outgroup is more important for a relevant reference group (i.e., social identification); and if the outgroup’s activities infringe strongly on the individual’s core values (i.e., values) [38].

Using concrete examples, even if both attitudes are perhaps negative in valance, attitudes towards persons who are affected by disabilities are expected to be less important than attitudes towards others who have committed a crime. A person’s disability typically does not inflict restrictions on the lifestyle of the general public. Committing a crime, on the contrary, directly and indirectly affects third parties and can have severe negative implications for the quality of life in a neighborhood. Moreover, committing a crime violates universal moral values, core values of different religious communities, as well as the law. Indeed, the public in the United Kingdom (UK), where the present research was conducted, indicated in two recent polls that ‘crime’ was one of the five most important issues facing the country ( [45]; the economy, health, immigration, and the environment were viewed as more important (see also Office for National Statistics (ONS) [46]).

If attitudes towards individuals who have committed a crime are attributed a higher significance than attitudes about people with a disability, the former should be less likely to change even if one is exposed to an intervention that aims to reduce stigmatization. Therefore, the aforementioned effects of outgroup descriptions that rely on person-first language (see *Hypothesis 1a -1e)* are expected to be smaller for the outgroup “people who have committed a violent crime” than “people who have a physical disability” (*Hypothesis 2*).

## Method

All data, analytical scripts, and materials as well as all Supplementary Materials are available on this Open Science Framework registry https://osf.io/u54k8/?view_only=66cd4d8abfd94c588603abece4a2afd9. The departmental ethics committee of the Department of Security and Crime Science, UCL had granted written ethical approval for this study. Participants provided informed consent, including consent to sharing their data, by ticking a box in an online form. Two participants did not agree to share their data from the main study; these data points were removed from the public dataset. Four participants did not agree to share their data from the pre-test; these data points were also removed from the public dataset.

### Design

To assess the hypotheses, we conducted a single-blind (online) experiment. Participants did not know to which condition they had been assigned but researchers were aware of the respective treatments when analyzing the data. We applied a between-subjects design that included two factors (target group x descriptor) with two levels respectively (target group: people who have a physical disability, people who have committed a violent crime; descriptor: identity-first, person-first) as well as a control group. Simple randomization was implemented to assign participants to conditions.

### Data collection procedure

Data was collected through the online opt-in access panel Prolific Academic. Participants self-identified by responding to an invitation on the platform and received a reimbursement of 0.77 GBP. A pre-test, which captured attitude importance, was conducted one week before the main study. Unfortunately, not all participants returned to the study after having done the pre-test. The pre-test was completed by *N* = 725 participants, 44 of whom dropped out of the study. This attrition rate is acceptable.

### Sample

We recruited participants until 681, the pre-registered planned sample size, had completed both the pre-test and the main study. The sample size calculation was based on an a-priori power analysis, considering the smallest effect of interest as a reference [47]. Our goal was to obtain.95 power, with α = .01 (Bonferroni-corrected p-value for five outcomes; see below), to be able to detect also small effects (or differences) of f = .175 with univariate analyses of variance (one-way) (i.e., the main analysis). We only recruited participants who were UK residents and older than 18 years. We introduced the former selection criterion (i.e., being a UK resident) as we applied definitions of disability and violent crime (see Manipulated variables – experimental stimuli) provided by UK institutions.

Participants (of the main study) were on average *M* = 42.6 (*SD* = 12.8; range: 18–79) years old. The majority of participants were female (60%), 38% were male, 0.7% indicated their gender identity as non-binary, and 0.8% of participants preferred not to answer this question. Participants varied in terms of their educational background. However, most participants had an advanced degree (55.7% Bachelor or Master’s degree, 3.5% PhD), 21.6% had completed their A levels, 18.6% reported having GCSE, O levels, or CSEs, and only 0.6% of participants had no qualifications. A total of 89.4% of participants described their ethnicity as White, followed by 5.4% who reported their ethnicity as Asian or Asian British, 2.9% who indicated Mixed ethnicity, 1.6% of participants who noted Black or Black British ethnicity, and 0.1% of participants who belonged to an Other ethnicity (0.4% preferred not to respond to this question). Somewhat unexpectedly, 3.2% of participants highlighted that they had committed a violent crime in the past. In addition, 8.5% of participants denoted that they describe themselves as a person with a physical disability.

### Variables

#### Manipulated variables – experimental stimuli.

In line with previous research [11,12], the experimental conditions (and the control condition) were defined by referring to (one of) five different groups when presenting the outcome measures (Table 1). That is, participants completed outcome measures considering either ‘most people’ (control group), ‘violent criminals’ (identity-first language), ‘people who have committed a violent crime’ (person-first language), ‘the physically disabled’ (identity-first language), or ‘people who have a physical disability‘ (person-first language).

**Table 1:** Instructions and group definitions.

Condition	Instructions and definition
Control group	To what extent do you agree with the following statements about most people?
Negative attitudes are expected to be more important, identity-first language	To what extent do you agree with the following statements about violent criminals? Definition of a violent crime: harmful force is used upon a victim, with or without using a weapon, ranging from common assault to murder (CPS, 2023)
Negative attitudes are expected to be more important, person-first language	To what extent do you agree with the following statements about people who have committed a violent crime? Definition of a violent crime: harmful force is used upon a victim, with or without using a weapon, ranging from common assault to murder (CPS, 2023)
Negative attitudes are expected to be less important, identity-first language	To what extent do you agree with the following statements about the physically disabled? Definition of physical disability: a physical impairment that has a ‘substantial’ and ‘long-term’ negative effect on the ability to do normal daily activities (UK Equality Act 2010)
Negative attitudes are expected to be less important, person-first language	To what extent do you agree with the following statements about people with a physical disability? Definition of physical disability: a physical impairment that has a ‘substantial’ and ‘long-term’ negative effect on the ability to do normal daily activities (UK Equality Act 2010)

#### Measured variables.

*Attitude importance* was measured in a pre-test. Attitude importance is commonly assessed by asking participants how concerned they are about a specific issue, rather than how much they care about their attitudes on this issue; both approaches have been found to be valid [15]. Participants were presented with a list of five topics (i.e., two target and three filler topics: violent crime, taxes, physical disability, climate change, and product placement in movies) and were asked to indicate their opinion about the topics (i.e., valence; 1 = very negative, 7 = very positive) as well as how important these topics were for them (1 = not at all important, 5 = very important). That is, in the pre-test, we referred neither to person- nor to identity-first language.

Unless indicated otherwise, all measures mentioned below applied a 5-point Likert-type scale (1 = ‘not at all’, 3 = ‘neither agree nor disagree’, 5 = ‘completely’). In the main study, and after providing participants with a definition of either the term physical disability or violent crime (Table 1), we employed an adapted version of Enock and Over’s [48] scales that explore desirable and undesirable outgroup traits that are either uniquely human (i.e., two positive stereotypes: knowledgeable, open-minded; two negative stereotypes: arrogant, controlling) or traits that humans share with animals (i.e., dehumanization: calm, curious, inflexible, unsophisticated). In doing so, we distinguished stereotyping from dehumanization [48]. Negative affect was measured with three items (‘I would be afraid to be around ….’, ‘I would be upset if … moved into my neighbourhood’, ‘I feel disgusted by …’). Outgroup approach intentions examined willingness for intergroup contact in different settings (‘I would not want to work with …’, ‘I would not want to live near …’, ‘I would want to be friends with …’, ‘I would never be willing to date …’).

An attention check was embedded in the list of aforementioned items (‘This is an attention check. Please answer ‘Not at all’ to indicate that you pay attention.’). Lastly, participants were asked to complete a manipulation check (‘You have just answered several questions about a specific group of people. How was this group referred to?’ Answer options: Violent criminals, People with a physical disability, Most people, The physically disabled, People who have committed a violent crime, My friends) and indicate whether they themselves have ever committed a violent crime (No/Yes) or described themselves as a person with a physical disability (No/Yes).

#### Indices.

The following indices were created by calculating mean scores: positive traits that are uniquely human (positive stereotypes (α = .76), higher values indicate stronger positive stereotyping); negative traits that are uniquely human (negative stereotypes (α = .86), higher values indicate stronger negative stereotyping); positive and negative traits shared with animals (dehumanization (α = .69), the items calm and curious were reverse-coded, higher values indicate stronger dehumanization); negative affect (α = .94, higher values indicate stronger negative affect); as well as outgroup approach intentions (α = .91, higher values indicate *lower* approach intentions).

## Results

### Randomization

There was no significant difference between the experimental conditions regarding the valence and importance of the topics of violent crime or physical disability as assessed in the pre-test, thus, prior to exposure to the experimental stimuli (Table 2).

**Table 2:** Between-group differences of pre-test measures of topic valence and importance.

Variable	ANOVA result
Valence violent crime	*F*(4, 663) =.27, *p = *.900, *η*^*2*^ = .00
Valence physical disability	*F*(4, 663) =.78, *p = *.536, *η*^*2*^ = .01
Importance violent crime	*F*(4, 663) = 2.35, *p = *.053, *η*^*2*^ = .01
Importance physical disability	*F*(4, 663) = 1.78, *p = *.130, *η*^*2*^ = .01

### Data exclusion

We had pre-registered that we would exclude data from all participants who did not fully complete the study, failed either the attention or the manipulation check, or who indicated that they had ever committed a violent crime or described themselves as a person with a physical disability. All participants completed the study fully. However, four participants failed the attention check. These were removed from all subsequent analyses.

Table 3 further indicates that several participants did not recall the precise wording of how the respective social groups were described (i.e., either identity- or person-first language). Many of these participants were, however, aware of which social group was discussed more generally. For instance, a substantial number of participants who were exposed to the descriptor “people who have committed a violent crime” reported that they answered questions regarding “violent criminals”. This result is further explored in the Supplementary Materials (S1 File) as it could suggest that certain individuals find it harder to process and, thus, recall person-first language. We removed all 95 cases that had failed the manipulation check.

**Table 3:** Outcomes of the manipulation check.

Condition	Manipulation check answer					Total
	Violent criminals	People with a physical disability	Most people	The physically disabled	People who have committed a violent crime	
Violent criminals	136	0	0	0	0	136
People who have committed a violent crime	47	0	0	0	89	136
Control	1	1	133	0	2	137
People with a physical disability	0	126	0	8	0	134
The physically disabled	0	36	0	102	0	138
Total	184	163	133	110	91	681

Lastly, a total of 71 participants reported that they had either committed a violent crime in the past, that they described themselves as a person with a physical disability, or that both applied to them. Data from these participants was also removed from further analyses. Below, we report results for the sample (*N *= 522) where all aforementioned data exclusion rules were followed as specified in the pre-registration. As the exclusions did not affect all conditions equally, sub-samples of the experimental conditions varied in size in the analytical sample. We considered this fact in the choice of (robust) statistical tests. Additionally, as the exclusions were overall substantial, we also document in the Supplementary Material (S2 File) hypotheses tests pertaining to a (larger) sample (*N* = 673) where data exclusion was less conservative and differed from the original data plan. Result patterns were largely the same; observed discrepancies are noted in the Supplementary Material (S2 File).

### Missing data

There were no missing data in the dataset.

### Outliers

We calculated the median absolute deviation to detect potential univariate outliers for all outcome variables [49]. Results showed that outliers were only identified for the variable ‘valence violent crime’ (Supplementary Materials S3 File). As pre-registered, we did not exclude outliers.

### Descriptive statistics

Mean scores and standard variations (for the full sample) as well as bi-variate correlations between all outcome measures are presented in Table 4. Table 5 further highlights mean scores and standard deviations of the dependent variables in each experimental condition. Bi-variate correlations in these five sub-samples are outlined in the Supplementary Material (S4 File).

**Table 4:** Descriptive statistics (full sample).

	Variable	*M* (*SD*)	1	2	3	4	5
	Valence violent crime (*N *= 725)	1.50 (1.00)					
	Valence physical disability (*N *= 725)	4.22 (1.37)					
	Importance violent crime (*N *= 725)	3.64 (1.12)					
	Importance physical disability (*N *= 725)	3.27 (1.15)					
1	Positive stereotypes (*N = *522)	3.11 (.83)	1				
2	Negative stereotypes (*N = *522)	2.67 (1.16)	−.58	1			
3	Dehumanization (*N = *522)	2.85 (.77)	−.71	.74	1		
4	Negative affect (*N = *522)	2.50 (1.47)	−.56	.83	.74	1	
5	Approach intentions (*N = *522)	3.04 (1.35)	−.61	.79	.74	.89	1

*Note.* All correlations are statistically significant at *p *< .001

**Table 5:** Mean scores and standard deviations of dependent measures across experimental conditions.

Variable	Control *M* (*SD*) *N *= 119	Violent criminals *M* (*SD*) *N *= 128	People who have committed a violent crime *M* (*SD*) *N *= 77	The physically disabled *M* (*SD*) *N *= 86	People with a physical disability *M* (*SD*) *N *= 112
Positive stereotypes	3.13 (.76)	2.63 (.68)	2.58 (.58)	3.79 (.73)	3.49 (.70)
Negative stereotypes	2.34 (.67)	3.88 (.71)	3.49 (.78)	1.65 (.74)	1.85 (.82)
Dehumanization	2.69 (.57)	3.50 (.61)	3.40 (.61)	2.21 (.57)	2.38 (.46)
Negative affect	1.91 (.61)	4.25 (.69)	3.86 (.88)	1.19 (.47)	1.18 (.41)
Approach intentions	2.92 (.87)	4.50 (.64)	4.08 (.77)	1.71 (.68)	1.79 (.60)

### Hypothesis tests – Confirmatory analyses

We first examined the impact of exposure to person-first as compared to identity-first language for the descriptor pairing “people with a physical disability/the physically disabled” (Hypothesis 1a – 1e). The Levene’s test as well as Q-Q plots suggested that the pre-registered two-tailed univariate analysis of variances (ANOVAs) were suitable for the outcome ‘positive stereotypes’ (*F*(2, 314) =.88, *p* = .414) and ‘dehumanization’ (*F*(2, 314) = 2.49, *p* = .084) but not the dependent variables ‘negative stereotypes’ (F(2, 314) = 6.31, *p* = .002), ‘negative affect’ (*F*(2, 314) = 12.12, *p* < .001) and ‘approach intentions’ (*F*(2, 314) = 10.42, *p* < .001). Kruskal-Wallis tests were computed for those latter three measures. Following, two planned contrasts (or Mann-Whitney U tests as non-parametric alternatives) assessed a) whether average negative outgroup perceptions and affect are lower, positive perceptions and approach intentions are higher in the person-first as compared to the identity-first language condition as well as b) whether negative outgroup perceptions and affect are lowest as well as positive perceptions and approach intentions are highest in the control condition.

Results showed that for the descriptor pairing “the physically disabled/people with a physical disability”, exposure to person- as compared to identity-first language slightly *reduced* positive stereotypes (rejecting Hypothesis 1d) and somewhat *increased* dehumanization (rejecting Hypothesis 1e). Person-first language did not affect the dependent variables ‘negative stereotypes‘, ‘negative affect‘, and ‘approach intentions‘ (rejecting Hypotheses 1a, 1b, 1c; Table 6). Moreover, other than expected, “most people” (i.e., the control condition) were viewed *less* positively and dehumanized *more* strongly than “the physically disabled/people with a physical disability”.

**Table 6:** ANOVA results and planned contrasts for all outcomes; descriptor pairing “the physically disabled/people with a physical disability”.

Outcome	ANOVA	Contrast a	Contrast b
Positive stereotypes	*F*(2, 314) = 20.77, *p *< .001, *η*^*2*^ = .12	*t*(314) = −2.85, *p *= .005, mean difference CI95[−.51, −.09]	*t*(314) = −5.99, *p < *.001, mean difference CI95[−1.36, −.69]
Dehumanization	*F*(2, 314) = 20.19, *p *< .001, *η*^*2*^ = .12	*t*(314) = −2.20, *p *= .029, mean difference CI95[−.32, −.02]	*t*(314) = −42.05, *p < *.001, mean difference CI95[−1.04, −.56]
**Outcome**	**Kruskal-Wallis Test**	**Mann-Whitney U Test a**	**Mann-Whitney U Test b**
Negative stereotypes	*H*(2) = 44.38, *p *< .001	*W* = 5448.5, *p *= .951, rank biserial correlation CI95[- ∞ ,.26]	**1** (p > c) *W* = 8946.5, *p *= 1.00, rank biserial correlation CI95[- ∞ ,.45] **2** (i > c) *W* = 7763.00, *p =* 1.00, rank biserial correlation CI95[- ∞ ,.61]
Negative affect	*H*(2) = 127.45, *p *< .001	*W* = 4867, *p *= .569, rank biserial correlation CI95[- ∞ ,.15]	**1** (p > c) *W* = 11326, *p *= 1.00, rank biserial correlation CI95[- ∞ ,.76] **2** (i > c) *W* = 8664, *p *= 1.00, rank biserial correlation CI95[- ∞ ,.76]
Approach intentions	*H*(2) = 115.52, *p *< .001	*W* = 5336.5, *p *= .906, rank biserial correlation CI95[- ∞ ,.24]	**1** (p > c) *W* = 11376, *p *= 1.00, rank biserial correlation CI95[- ∞ ,.76] **2** (i > c) *W* = 8828.5, *p *= 1.00, rank biserial correlation CI95[- ∞ ,.78]

*Note.* 1 (p > c): values expected to be higher in person-first language than control condition; 2 (i > c): values expected to be higher in identity-first language than control condition

The aforementioned analyses were repeated for the descriptor pairing “people who have committed a violent crime/violent criminals”. The Levene’s test, as well as the Q-Q plots, suggested that two-tailed ANOVAs were suitable for the outcomes ‘negative stereotypes’ (*F*(2, 321) =.96, *p *= .383) and ‘dehumanization’ (*F*(2, 321) =.228, *p *= .796) but not the dependent variables ‘positive stereotypes’ (*F*(2, 321) = 3.86, *p *= .022), ‘negative affect’ (*F*(2, 321) = 9.04, *p *< .001), and ‘approach intentions’ (*F*(2, 321) = 8.20, *p *< .001). Kruskal-Wallis tests were computed for those latter three measures. As previously, these tests were followed by planned contrasts or Mann-Whitney U tests.

Results in Table 7 indicate that the proposed beneficial effects of person-first language were confirmed with regards to (reduced) ‘negative stereotypes’ (confirming Hypothesis 1a) as well as (reduced) ‘negative affect’ (confirming Hypothesis 1b) and (increased) ‘approach intentions’ (confirming Hypothesis 1c) but not (increased) ‘positive stereotypes’ and (lower) ‘dehumanization’ (rejecting Hypothesis 1d and 1e). Furthermore, as expected, perceptions, attitudes, and behavioral intentions were overall less positive or more negative in either experimental condition compared to the control condition.

**Table 7:** ANOVA results and planned contrasts for all outcomes; descriptor pairing “violent criminals/people who have committed a violent crime”.

Outcome	ANOVA	Contrast a	Contrast b
Negative stereotypes	*F*(2, 321) = 151.75, *p *< .001, *η*^*2*^ = .49	*t*(321) = 3.79, *p *< .001, mean difference CI95[.19,.59, −.22]	*t*(321) = 16.22, *p *< .001, mean difference CI95[2.37, 3.02]
Dehumanization	*F*(2, 321) = 63.85, *p *< .001, *η*^*2*^ = .29	*t*(321) = 1.20, *p = *.232, mean difference CI95[−.07,.27]	*t*(321) = 10.92, *p < *.001, mean difference CI95[1.24, 1.79]
**Outcome**	**Kruskal-Wallis Test**	**Mann-Whitney U Test a**	**Mann-Whitney U Test b**
Positive stereotypes	*H*(2) = 39.22, *p *< .001	*W* = 5133.50, *p = *.701, rank biserial correlation CI95[- ∞ ,.18]	**1** (p < c) *W* = 2600, *p < *.001, rank biserial correlation CI95[- ∞ , −.31] **2** (i < c) *W* = 4674.50, *p < *.001, rank biserial correlation CI95[- ∞ , −.28]
Negative affect	*H*(2) = 210.17, *p *< .001	*W* = 6184.00, *p < *.001, rank biserial correlation CI95[.12, ∞]	**1** (p > c) *W* = 8729, *p < *.001, rank biserial correlation CI95[.88, ∞] **2** (i > c) *W* = 15057, *p < *.001, rank biserial correlation CI95[.97, ∞]
Approach intentions	*H*(2) = 149.99, *p *< .001	*W* = 6640.50, *p < *.001, rank biserial correlation CI95[.22, ∞]	**1** (p > c) *W* = 7651.50, *p < *.001, rank biserial correlation CI95[.59, ∞] **2** (i > c) *W* = 14035.50, *p < *.001, rank biserial correlation CI95[.80, ∞]

*Note.* 1 (p > c): values expected to be higher in person-first language than control condition; 2 (i > c): values expected to be higher in identity-first language than control condition

To investigate Hypothesis 2, that is, that the effect of person-first language is stronger for social groups about which individuals hold more important (negative) attitudes, we sought to verify first that participants did, indeed, hold largely negative views on the topics of violent crime and physical disability and that the former topic was considered more important. Information on attitude valence and importance was collected in the pre-test; these analyses are, therefore, based on *N* = 725 participants. The Shapiro-Wilk test indicated that assumptions of normal distribution of the outcome variables ‘valence violent crime’ (*W* = .56, *p* < .001), ‘valence physical disability’ (*W* = .91, *p* < .001), ‘importance violent crime’ (*W* = .89, *p* < .001), and ‘importance physical disability’ (*W *= .91, *p *< .001) were not fulfilled. Hence, we conducted one-tailed Wilcoxon signed-rank tests.

As postulated, attitudes about violent crime were strongly negative (i.e., testing whether the variables’ mean score was below 3; *M* = 1.50, *SD* = 1.00; *W* = 13023, *p* < .001, rank biserial correlation CI95[- ∞ , −.88]). The same cannot be said about attitudes towards physical disability (*M* = 4.22, *SD* = 1.37; *W* = 193149, *p* = 1.00, rank biserial correlation CI95[- ∞ , .81]). An exploratory one-sided Wilcoxon signed-rank test with the reverse alternative hypothesis highlighted that, instead, attitudes towards physical disability were highly positive (*W* = 193149, *p* < .001, rank biserial correlation CI95[.76, ∞]). Indeed, participants rated the topic of physical disability significantly more positive than that of violent crime (*W* = 1840.5, *p* < .001, rank biserial correlation CI95[- ∞ , −.98]).

Next, we tested whether attitudes towards violent crime were more important than those towards physical disability. The Wilcoxon signed-rank test demonstrated that in line with our expectation attitudes towards violent crime (*M* = 3.64, *SD* = 1.12) were considered more important than those towards physical disability (*M* = 3.27, *SD* = 1.15; *W* = 68249, *p* < .001, rank biserial correlation CI95[.32,∞]). In summary, as physical disability was viewed as strongly positive, the two social groups that were presented in the descriptor pairings differed in terms of valence *and* attitude importance.

We had pre-registered two-sided equivalence tests for each outcome variable to assess whether the effect of person-first language was stronger for the descriptor pairing “violent criminals/people who have committed a violent crime”. However, in the latter sub-sample, statistically significant influences of person-first language were confirmed for three outcomes (i.e., ‘negative stereotypes’, ‘negative affect’, ‘approach intentions’), while no significant differences in mean scores on the same measures were identified in the sub-sample that reported views on either "the physically disabled" or "people with a physical disability". Thus, Hypothesis 2, examining the moderating role of only attitude importance, could not be tested as intended.

### Exploratory analysis

**Fig 1:**
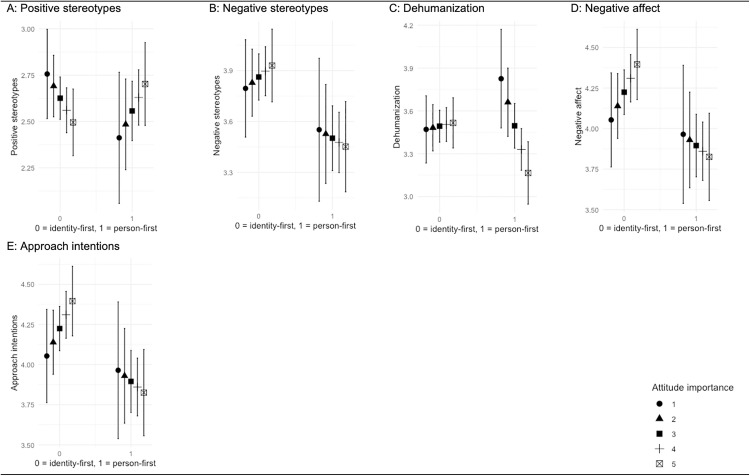
Marginal Effects Plots Descriptor Pairing “violent criminals/people who have committed a violent crime”.

An alternative analytical approach was chosen to provide, nonetheless, tentative insights about the moderating role of attitude importance on the effects of person-first language. Interaction effects between attitude importance as reported in the pre-test and exposure to identity-first or person-first language were assessed for all five outcome variables, separately for each descriptor pairing. Results (Table 8) showed that for the descriptor pairing “violent criminals/people who have committed a violent crime” the interaction term was not significant for the outcomes ‘positive stereotypes’ (*F*(3, 195) = 1.13, *p = *.339, *R*^*2*^ = .00), ‘negative stereotypes’ (*F*(3, 195) = 4.53, *p = *.004, *R*^*2*^ = .05), ‘negative affect’ (*F*(3, 195) = 5.04, *p = *.002, *R*^*2*^ = .06), and ‘approach intentions’ (*F*(3, 195) = 6.80, *p* *< *.001, *R*^*2*^ = .08). For the dependent variable ‘dehumanization’, a small moderating effect of attitude importance was, however, documented (*F*(3, 195) = 2.87, *p = *.038, *R*^*2*^ = .03). Inspection of the marginal effects (Fig 1, Panel C) indicates that within the condition of person-first language, those who attributed higher importance to the topic of violent crime reported lower dehumanization. Nonetheless, and speaking more directly to the rationale of our hypothesis that stipulates weaker effects of person-first language for participants who consider violent crime more important, between-group comparisons did not demonstrate that the strength of the impact of exposure to person-first (vs. identity-first) language on dehumanization varied between participants who assigned weaker or stronger significance to violent crime. Conversely, although the respective interaction terms were not statistically significant, marginal effects plots suggested such between-group differences for the outcomes ‘negative stereotypes’ (Fig 1, Panel B), ‘negative affect’ (Fig 1, Panel D), and ‘approach intentions’ (Fig 1, Panel E). Noting that these patterns must be interpreted very carefully, they would contest our hypothesis; for each of these dependent variables, exposure to person-first (vs. identity-first) language had a larger (albeit still weak), beneficial effect for participants who held stronger attitudes on violent crime.

**Table 8:** Assessing interaction effects of attitude importance to explore Hypothesis 2.

Descriptor pairing “violent criminals/people who have committed a violent crime”					
Predictor	Positive stereotypes	Negative stereotypes	Dehumanization	Negative affect	Approach intentions
Experimental condition	*B* = −.48 (.29), *p = *.098	*B* = −.19 (.35), *p = *.593	*B* = .53 (.28), *p = *.061	*B* = .03 (.35), *p = *.928	*B* = −.01 (.31), *p = *.972
Attitude importance	*B* = −.07 (.05), *p = *.153	*B* = .03 (.05), *p = *.534	*B* = .01 (.04), *p = *.797	*B* = .09 (.05), *p = *.120	*B* = .04 (.05), *p = *.463
Interaction term	*B* = .14 (.08), *p = *.078	*B* = −.06 (.09), *p = *.526	*B* = −.18 (.08), *p = *.021	*B* = −.12 (.09), *p = *.200	*B* = −.12 (.08), *p = *.170
**Descriptor pairing “the physically disabled/people with a physical disability”**					
Predictor	Positive stereotypes	Negative stereotypes	Dehumanization	Negative affect	Approach intentions
Experimental condition	*B* = .11 (.32), *p = *.725	*B* = .34 (.36), *p = *.349	*B* = .43 (.23), *p = *.064	*B* = −.37 (.15), *p = *.070	*B* = −.26 (.29), *p = *.363
Attitude importance	*B* = .23 (.07), *p = *.002	*B* = −.15 (.08), *p = *.057	*B* = −.04 (.05), *p = *.443	*B* = −.13 (.04), *p = *.005	*B* = −.18 (.06), *p *= .005
Interaction term	*B* = −.13 (.10), *p = *.187	*B* = −.03 (.11), *p = *.755	*B* = −.08 (.07), *p = *.237	*B* = .11 (.06), *p = *.056	*B* = .09 (.08), *p = *.246

*Note*. unstandardized coefficients are presented; standard errors are presented in brackets

**Fig 2:**
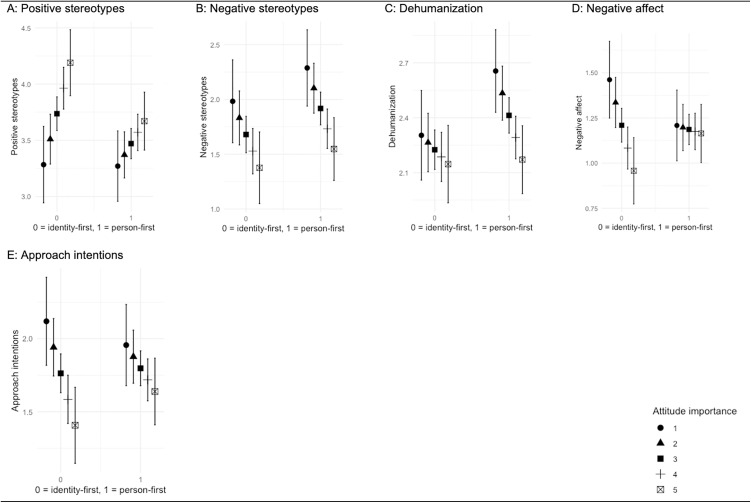
Marginal Effects Plots Descriptor Pairing “the physically disabled/people with a physical disability”.

We repeated the aforementioned analyses for the descriptor pairing “the physically disabled/people with a physical disability”. Results showed no significant interaction term (‘positive stereotypes’: *F*(3, 191) = 6.90, *p* *< *.001, *R*^*2*^ = .08; ‘negative stereotypes’: *F*(3, 191) = 4.77, *p = *.003, *R*^*2*^ = .06; ‘dehumanization’: *F*(3, 191) = 4.23, *p = *.006, *R*^*2*^ = .05; ‘approach intentions’: *F*(3, 191) = 3.41, *p *= .019, *R*^*2*^ = .04, ‘negative affect’: *F*(3, 191) = 2.71, *p = *.046, *R*^*2*^ = .03). Marginal effects plots also did not point to between-group differences, that is, the strength of the impact of exposure to person-first (vs. identity first) language in this context did not vary between participants who attributed weaker or stronger importance to the topic of physical disability (Fig 2; Panel A–E).

## Discussion

Person-first language is encouraged across different settings [1–5]. However, to date, only a small number of empirical studies have confirmed its proposed benefits, that is, that exposure to person-first language reduces outgroup stigmatization [9–12]. Importantly, it has not been established whether the positive impact of person-first language can be generalized across (stigmatized) social groups. This registered report advanced the literature and assessed the effects of exposure to person-first language with respect to two social groups. We further examined the moderating role of attitude importance [16,38]. More precisely, we sought to investigate if the influence of exposure to person-first language on outgroup perceptions, attitudes, and approach intentions is weaker when considering a group towards which negative attitudes are more (as compared to less) important.

Results supported our hypotheses only partially. We did replicate previous research [10–12] for the descriptor pairing “violent criminals/people who have committed a violent crime” and showed that exposure to person-first language reduced negative stereotypes and affect as well as increased approach intentions. Demonstrating that approach intentions were enhanced is especially promising as work on intergroup contact has highlighted that such interactions could ultimately reduce prejudice [50], thereby multiplying the impact of person-first language. Effect sizes were small to moderate and, thus, weaker than in Baker and colleagues’ [10] and Granello and Gibbs’ [14] work. Nonetheless, these findings emphasize the added value of describing individuals who have committed a violent crime using person-first terminology.

Having said this, the underlying mechanisms of the effects remain unclear. It is argued that person-first language highlights an individual’s personhood and individuality rather than certain stigmatized attributes [6,7]. However, we demonstrated that referring to someone as a person who has committed a violent crime rather than a violent criminal did *not* reduce average levels of dehumanization. The finding may imply that person-first language did, in fact, not contribute to enhanced notions of personhood. Our tentative interpretation should be confirmed in future studies that use alternative measures of dehumanization. We captured traits of human uniqueness, and measures of implicit or blatant dehumanization [51] may be better suited to investigate mechanisms that drive the effects of person-first language.

It is also worth noting that reported levels of positive stereotyping did not differ significantly between participants who were exposed to person- or identity-first language for the descriptor pairing “violent criminals/people who have committed a violent crime”. Positive stereotypes were low across the identity- and person-first language conditions, such that ceiling effects cannot explain the pattern (see below). The result may instead point to the role of negativity bias [52]. More precisely, negative information about an object or person tends to be more salient and attract more attention than positive information [52] (e.g., for people who have committed violent crimes, negative traits may be more salient than positive traits). In turn, more salient (negative) perceptions are more susceptible to change or are perhaps initially changed more strongly than positive attributes. Therefore, we carefully conclude a potential distinction in how person-first terminology influences positive and negative outgroup perceptions.

Although we documented beneficial effects of person-first language for one descriptor pairing, referring to a group as “people with a physical disability” rather than “the physically disabled” had no impact on negative outgroup perceptions, affect, or approach intentions but decreased positive stereotypes and enhanced dehumanization slightly. This result contests previous studies that have generally highlighted that exposure to person-first terminology would contribute to more favorable views of individuals affected by different medical conditions [10,13,14]. The outcome is perhaps even more surprising when considering that participants’ attitudes towards physical disability were less important but also substantially positive.

Ceiling effects could explain the nil findings. In other words, if opinions of people with a physical disability are already strongly positive, there remains little scope for person-first language to improve negative views. In such a scenario, exposure to identity-first language, which might signal stigmatization to participants, may also have triggered a backfire effect, a defensive reaction that emboldened positive views about people with a physical disability. Alternatively, person-first terminology, rather than highlighting personhood, has been shown to serve as a signal for the common stigmatization of the group [8] (i.e., implying that the group faces widespread stigma, thus necessitating person-first language) and could thereby inadvertently undermine positive outgroup perceptions [20,21]. Lastly, as noted, person-first terminology aims to avoid essentialism and seeks to highlight that stigmatized attributes can be changed [6]. Certain characteristics are more easily mutable or are perceived to be more easily mutable than others. Attributes that are viewed as more *im*mutable likely evoke stronger essentialist thinking, which is associated with a stronger endorsement of respective stereotypes [53]. Thus, person-first terminology may attain more benefits for social groups characterized by a stigmatized attribute that is seen as more mutable. The latter apply perhaps more readily to behavior, including criminal behavior, but not to certain medical conditions like having a physical disability. To investigate these different interpretations, future research should focus on identifying *why* exposure to person-first language shapes thoughts, feelings, and behaviors of third parties as well as whether the same mechanisms apply to different social groups.

A central aim of this study was to examine whether the influence of person-first terminology is weaker when referring to social groups towards which people hold more important negative attitudes. The results discussed above emphasize that, other than expected, the beneficial effects of person-first language were documented for the group towards which attitudes were negative and more, not less, important. Given that participants held strongly positive opinions about the topic of physical disability, we were not able to test our hypothesis regarding the moderating role of attitude importance as planned.

Having said this, exploratory analyses allow careful speculations. For the descriptor pairing “violent criminals/people who have committed a violent crime” – a topic towards which attitudes were negative and important – inspection of the marginal effects across all outcome measures appeared to suggest that higher levels of attitude importance encourage participants to be open to adjusting their outgroup perceptions and attitudes slightly. Given that crime is widely viewed as an important topic in the general population [45,46], the use of person-first terminology seems, once again, advisable when referring to individuals who have committed a violent crime. However, two caveats must be noted. First, the patterns depicted in the marginal effects plots were not confirmed by statistically significant interaction terms. Second, results pertaining to the descriptor pairing “the physically disabled/people with a physical disability” did not replicate the aforementioned trends.

Taken together, based on exploratory analyses, we do not find convincing evidence that attitude importance modulates the size of the effect of exposure to person-first language. We believe further elaboration of this question should apply a more robust design that allows the disentangling of dynamics of attitude importance from those of valence [31]. Ideally, outgroup attitude valence and importance would be actively manipulated prior to exposure to person-first language. More generally, we further conclude that public opinion polls are not necessarily a sufficiently exact guide of the perceived valence and importance of social groups in a particular study sample. Outgroup perceptions should ideally be assessed in a chosen population (e.g., UK residents on Prolific) before designing a study.

### Limitations

The conclusions that we have drawn in the previous paragraphs must be viewed in light of the following additional limitations. First, the study only compared the effects of exposure to person-first terminology used to describe two specific social groups. Without further conceptual replications, the findings should not be generalized to estimate the potential impact of person-first language per se, especially not regarding social groups towards which attitudes are negative and unimportant (see above). Moreover, although it could be speculated that attitudes about violent crime are negative and important across different demographic and cultural sub-samples, attitudes towards physical disability may vary more strongly. Our data was collected in the UK and the sample was, on average, highly educated. To what extent and how outgroup perceptions, attitudes, and behavioral intentions are affected when referring to “people with a physical disability” if data is collected in other settings or from other populations is unclear.

Importantly, similarly to most of the published literature, the present study focused only on examining the impact of exposure to person-first language on third-parties’ outgroup evaluations. Findings may not generalize to how members of the target groups themselves or professionals, such as health care providers or people working in the criminal justice system, respond to person-first language. Having said this, St. Louis [13] confirmed similar effects of person-first language across different stakeholders.

The result patterns further did not control for individual-level variables that either shape outgroup perceptions and behavioral intentions or could moderate the impact of exposure to person-first language. For instance, gender differences in explicit and implicit prejudice have been confirmed [54]. In turn, if participants were familiar with person-first language policies, and had perhaps already used person-first language regularly, the expected impact of the experimental manipulation would be lower (i.e., ceiling effect).

Finally, as discussed, a substantial number of participants failed the manipulation check and did not recall accurately that they had been exposed to person-first language. We considered this unexpected event an opportunity to explore whether the failure of recall attenuates the effect of person-first language, a possible reflection of cognitive dissonance. However, it is also worth noting that the latter might be an artifact and highlight a weakness of the experimental manipulation. We relied on previous studies to inform the design of the experimental manipulation [11,12]; item statements included, depending on the experimental condition, person- or identity-first descriptors (or the descriptor ‘most people’ in the control condition). We had hoped that the repeated use of the descriptors would increase their salience. Instead, though, it is possible that cognitive dissonance was evoked more strongly, as participants read the descriptor “people who committed a violent crime” a total of 15 times. To address this concern in future studies, vignettes could be used that employ only one instance of person- or identity-first language to describe different characters. Outcome measures should avoid repeating the descriptors. To improve ecological validity, the vignettes could be developed based on ‘real-world’ conversations or published media.

## Conclusion

Despite these challenges, we believe that this registered report makes important contributions to the literature. We demonstrated that exposure to person-first language improved the evaluation of a strongly stigmatized group, that is, people who have committed a violent crime. Having said this, our results challenge the assumption that the impact of person-first language can be generalized across social groups. Overall, we extend the discourse on person-first terminology by raising several open questions, hoping to inspire more empirical work that explores when and why person-first terminology reduces the stigmatization of a wide range of communities (also beyond the medical and criminal justice context).

In doing so, we assume that recognizing the worth and dignity of all human beings, preventing unjust discrimination, and avoiding the harms of dehumanizing language are uncontroversial goals; these are ‘the right things to do’. This aim may be criticized when it pertains to groups that engage in behaviors that cause harm to others, as in the case of people who have committed a violent crime. Some may worry that using person-first language in this instance could be seen as minimizing, normalizing, or condoning harmful conduct.

One key consideration in this context is whether improving attitudes towards a given group has wider societal benefits. Notably, there is evidence suggesting that reducing stigma and negative stereotypes towards individuals who have committed a criminal offense can contribute to lower recidivism rates and better reintegration outcomes [55–58], for example, by increasing engagement in rehabilitation programs [55]. Thus, there is a compelling consequentialist argument for using person-first language because reduced stigmatization has long-term positive impacts on society as a whole.

## Supporting Information

S1 File Comparison of participants who did or did not accurately recall exposure to person-first language. (DOCX)

S2 File Effects of person-first language across social groups. (DOCX)

S3 File Univariate outliers. (DOCX)

S4 File Bi-variate correlations of dependent variables in each experimental and the control condition. (DOCX)
