# Supplementary material for: Does attitude importance moderate the effects of person-first language? A registered report
Source: PLoS One. 2025 Oct 8;20(10):e0332733. doi: 10.1371/journal.pone.0332733 (PMC12507193; doi:10.1371/journal.pone.0332733)
Supplement: S1 File — (DOCX) [file pone.0332733.s001.docx]

**Supplementary Material S1**

As illustrated in Table 2 in the main text, 55 participants who were exposed to person-first language reported in the manipulation check that they answered questions including identity-first language. The majority (*n* = 47) were exposed to the descriptor ‘people who have committed a violent crime’ (*n* = 8 read instructions pertaining to ‘people with a physical disability’). In this additional exploratory analysis, we examined whether the aforementioned sub-sample differed in their outgroup perceptions, affect, and approach intentions from participants who had recalled accurately that they had been exposed to person-first language. This speculation was confirmed. Findings demonstrated that those who accurately recalled that they were exposed to person-first language reported stronger positive stereotypes as well as lower negative stereotypes, dehumanization, negative affect, and higher outgroup approach intentions (Table S1.1).

*Table S1.1*

Comparison of participants who did or did not accurately recall exposure to person-first language

| **Outcome** | **Kruskal-Wallis Test** | **Accurately recalled exposure to person-first language**  ***M* (*SD*)** | **Did not accurately recall exposure to person-first language**  ***M* (*SD*)** |
| --- | --- | --- | --- |
| Positive stereotypes | *H*(1) = 7.11, *p =* .008 | 3.11 (.82) | 2.76 (.74) |
| Negative stereotypes | *H*(1) = 17.72, *p* < .001 | 3.51 (1.14) | 3.20 (.98) |
| Dehumanization | *H*(1) = 17.90, *p* < .001 | 2.78 (.74) | 3.21(.61) |
| Negative affect | *H*(1) = 33.78, *p* < .001 | 2.29 (1.48) | 3.74 (1.23) |
| Approach intentions | *H*(1) = 25.26, *p* < .001 | 2.72 (1.33) | 3.91 (1.13) |

Failure to recall exposure to person-first language may indicate that participants withdrew attention that would be required for systematic processing of the repetitive stimuli, that is, the descriptor, while focusing on the novel stimuli, that is, the remaining item text (i.e., habituation to the repeated distractor, [1]). One conceivable explanation is that for some individuals – those who held views that strongly reject the ‘humanness’ of “people who have committed a violent crime” – systematic processing of person-first terminology might elicit cognitive dissonance and reactance [2]. In other words, results may point to a backfire effect that is well-established for different communication-based strategies that seek to change attitudes, such as counter-narratives and attitudinal inoculation [3, 4]. Specifically, instead of reducing stigmatization, for some individuals, exposure to person-first language may substantially challenge their beliefs, and to cope with this threat, existing negative viewpoints are emboldened. This backfire effect is likely even further pronounced if individuals are required to use person-first language in their communications. Two avenues should be explored in future studies: does failure to recall exposure to person-first language indicate cognitive dissonance as well as who is especially prone to such backfire effects.

**References**

1. Turatto M. Habituation (of attentional capture) is not what you think it is. Journal of Experimental Psychology: Human Perception and Performance. 2023 Aug;49(8):1132.
2. Festinger L. *A theory of cognitive dissonance*. Evanstone, IL: Row, Peterson; 1957.
3. Bélanger JJ, Nisa CF, Schumpe BM, Gurmu T, Williams MJ, Putra IE. Do counter-narratives reduce support for ISIS? Yes, but not for their target audience. Frontiers in psychology. 2020 Jun 11;11:1059.
4. Schumann S, Barton M. Does Attitudinal Inoculation Confer Resistance to Violent Extremist Propaganda? Assessing Mechanisms, Long‐Term Effects, and the Advantage of Visuals. Journal of Community & Applied Social Psychology. 2024 Nov;34(6):e2898.
