# Supplementary material for: Does attitude importance moderate the effects of person-first language? A registered report
Source: PLoS One. 2025 Oct 8;20(10):e0332733. doi: 10.1371/journal.pone.0332733 (PMC12507193; doi:10.1371/journal.pone.0332733)
Supplement: S2 File — (DOCX) [file pone.0332733.s002.docx]

**Supplementary Material S2**

**Data exclusion**

We had pre-registered that we would exclude data from all participants who did not fully complete the study, failed either the attention or the manipulation check, or who indicated that they had ever committed a violent crime or described themselves as a person with a physical disability. All participants completed the study fully. However, four participants failed the attention check. These were removed from all subsequent analyses.

Analysis of the manipulation check showed that several participants did not recall the precise wording of how the respective social groups were described (i.e., either identity- or person-first language) but they were aware of which social group was discussed (Table 2 in main text). Here, we decided *not* to remove the cases characterized by this specific pattern of a failed manipulation check. Having said this, we removed four data points from all subsequent analyses referring to participants who were in the control condition but who stated answering questions about groups described using either identity- or person-first language.

A total of 71 participants reported that they had either committed a violent crime in the past or that they described themselves as a person with a physical disability. This constituted a much larger proportion of the sample than we had expected. Here, we decided *not* to remove these participants from further analyses. Below, we report results pertaining to this (larger) sample (*N* = 673) that differs from the original data plan.

**Hypothesis Tests**

We first examined the impact of exposure to person-first as compared to identity-first language for the descriptor pairing “people with a physical disability/the physically disabled” (Hypothesis 1a – 1e). The Levene’s tests as well as Q-Q plots suggested that the pre-registered two-tailed univariate analysis of variances (ANOVAs) were suitable for the outcome ‘positive stereotypes’ (*F*(2, 400) = 1.12, *p* = .327) but not the dependent variables ‘negative stereotypes’ (*F*(2, 400) = 3.90, *p* = .021), ‘dehumanization’ (*F*(2, 400) = 3.08, *p* = .047), ‘negative affect’ (*F*(2, 400) = 14.24, *p* < .001) and ‘approach intentions’ (*F*(2, 400) = 12.39, *p* < .001). Non-parametric Kruskal-Wallis tests were computed for those latter four measures. Following, two planned contrasts (or Mann-Whitney U tests as non-parametric alternatives) assessed whether a) average negative outgroup perceptions and affect are lower, positive perceptions and approach intentions are higher in the person-first as compared to the identity-first language condition as well as b) whether negative outgroup perceptions and affect are lowest as well as positive perceptions and approach intentions are highest in the control condition.

Results showed that for the descriptor pairing “the physically disabled/people with a physical disability”, exposure to person- as compared to identity-first language *reduced* positive stereotypes (rejecting Hypothesis 1d) and did not affect the remaining dependent variables (rejecting Hypotheses 1a, 1b, 1c, and 1e; Table S2.2). Additionally, other than expected, perceptions, attitudes, and behavioral intentions towards people with a physical disability and the physically disabled were not less positive than those towards ‘most people’ (i.e., the control condition). Table S2.1 suggests that ‘most people’ were viewed less positively than people with a physical disability and the physically disabled.

*Table S2.1*

Mean scores and standard deviations of dependent measures across experimental conditions

| **Variable** | **Control**  ***M* (*SD*)**  *N* = 132 | **Violent criminals *M* (*SD*)** *N* = 136 | **People who have committed a violent crime *M* (*SD*)**  *N* = 134 | **The physically disabled *M* (*SD*)**  *N* = 137 | **People with a physical disability *M* (*SD*)** *N* = 134 |
| --- | --- | --- | --- | --- | --- |
| Positive stereotypes | 3.10 (.77) | 2.66 (.68) | 2.58 (.67) | 3.79 (.71) | 3.50 (.68) |
| Negative stereotypes | 2.36 (.70) | 3.87 (.71) | 3.48 (.75) | 1.71 (.77) | 1.81 (.79) |
| Dehumanization | 2.70 (.58) | 3.49 (.60) | 3.38 (.60) | 2.25 (.55) | 2.36 (.45) |
| Negative affect | 1.93 (.63) | 4.24 (.68) | 3.98 (.83) | 1.19 (.48) | 1.18 (.42) |
| Approach intentions | 2.94 (.87) | 4.48 (.64) | 4.16 (.73) | 1.67 (.65) | 1.75 (.60) |

*Table S2.2*

Confirmatory hypotheses tests for the descriptor pairing “the physically disabled/people with a physical disability”

| **Outcome** | **ANOVA** | **Contrast a** | **Contrast b** |
| --- | --- | --- | --- |
| Positive stereotypes | *F*(2, 400) = 30.58, *p* < .001, *η^2^* = .13 | *t*(400) = -3.24, *p* = .001, mean difference CI95[-.46, -.11] | *t*(400) = -7.10, *p <* .001, mean difference CI95[-1.39, -.79] |
| **Outcome** | **Kruskal-Wallis Test** | **Mann-Whitney U Test a** | **Mann-Whitney U Test b** |
| Negative stereotypes | *H*(2) = 51.76, *p* < .001 | *W* = 9836.5, *p* = .857, rank biserial correlation CI95[-∞, .19] | **1** (p>c) *W* = 12243, *p* = 1.00, rank biserial correlation CI95[-∞, .48]  **2** (i>c) *W* = 13239, *p* = 1.00, rank biserial correlation CI95[-∞, .55] |
| Dehumanization | *H*(2) = 36.81, *p* < .001 | *W* = 10222.5, *p* = .950, rank biserial correlation CI95[-∞, .23] | **1** (p>c) *W* = 11657, *p* = 1.00, rank biserial correlation CI95[-∞, .42]  **2** (i >c) *W* = 12614, *p* = 1.00, rank biserial correlation CI95[-∞, .49] |
| Negative affect | *H*(2) = 159.84, *p* < .001 | *W* = 9250, *p* = .559, rank biserial correlation CI95[-∞, .12] | **1** (p>c) *W* = 15058, *p* = 1.00, rank biserial correlation CI95[-∞, .76]  **2** (i>c) *W* = 15409, *p* = 1.00, rank biserial correlation CI95[-∞, .76] |
| Approach intentions | *H*(2) = 150.09, *p* < .001 | *W* = 10139, *p* = .934, rank biserial correlation CI95[-∞, .22] | **1** (p>c) *W* = 15322, *p* = 1.00, rank biserial correlation CI95[-∞, .78]  **2** (i>c) *W* = 15879, *p* = 1.00, rank biserial correlation CI95[-∞, .80] |

*Note.* 1 (p>c): comparison: values expected to be higher in person-first language than in the control condition; 2 (i>c): comparison: values expected to be higher in identity-first language than in the control condition

The aforementioned analyses were repeated for the descriptor pairing “people who have committed a violent crime/violent criminals”. The Levene’s tests as well as the Q-Q plots suggested that two-tailed ANOVAs were suitable for the outcomes ‘positive stereotypes’ (*F*(2, 399) = 2.08, *p* = .127), ‘negative stereotypes’ (*F*(2, 399) = .12, *p* = .887), and ‘dehumanization’ (*F*(2, 399) = .005, *p* = .995) but not the dependent variables ‘negative affect’ (*F*(2, 399) = 6.34, *p* = .002) and ‘approach intentions’ (*F*(2, 399) = 8.05, *p* < .001). Kruskal-Wallis tests were computed for those latter two measures. As previously, these were followed by planned contrasts or Mann-Whitney U tests.

Results in Table S2.3 indicate that the proposed beneficial effects of person-first language were confirmed with regards to (reduced) ‘negative stereotypes’ and (reduced) ‘negative affect’ as well as (improved) ‘approach intentions’ (confirming Hypothesis 1a, 1b, and 1c) but not (increased) ‘positive stereotypes’ and (lower) ‘dehumanization’ (rejecting Hypothesis 1d and 1e). Furthermore, as expected, perceptions, attitudes, and behavioral intentions were overall less positive in either experimental condition as compared to the control condition.

**Comparison with the Analysis Presented in the Main Text**

Findings of both analyses differed with respect to only one outcome. In the analysis that relied on pre-registered exclusion criteria (presented in the main text), exposure to person- as compared to identity-first language increased dehumanization slightly for the descriptor pairing “the physically disabled/people with a physical disability”; the direction of the between-group difference was, however, the same in both analyses.

*Table S2.3*

Confirmatory hypotheses tests for the descriptor pairing “violent criminals/people who have committed a violent crime”

| **Outcome** | **ANOVA** | **Contrast a** | **Contrast b** |
| --- | --- | --- | --- |
| Positive stereotypes | *F*(2, 399) = 20.68, *p* < .001, *η^2^* = .09 | *t*(399) = -.97, *p* = .334, mean difference CI95[-.25, .09] | *t*(399) = 6.36, *p <* .001, mean difference CI95[.66, 1.25] |
| Negative stereotypes | *F*(2, 399) = 157.66, *p* < .001, *η^2^* = .44 | *t*(399) = 4.43, *p* < .001, mean difference CI95[.22,.56] | *t*(399) = 17.18, *p* < .001, mean difference CI95[2.34, 2.94] |
| Dehumanization | *F*(2, 399) = 69.67, *p* < .001, *η^2^* = .26 | *t*(399) = 1.58, *p =* .115, mean difference CI95[-.03,.26] | *t*(399) = 11.69, *p <* .001, mean difference CI95[1.22,1.72] |
| **Outcome** | **Kruskal-Wallis Test** | **Mann-Whitney U Test a** | **Mann-Whitney U Test b** |
| Negative affect | *H*(2) = 244.17, *p* < .001 | *W* = 10736, *p =* .005, rank biserial correlation CI95[.06, ∞] | **1** (p>c) *W* = 17010, *p <* .001, rank biserial correlation CI95[.90, ∞]  **2** (i>c) *W* = 17696, *p <* .001, rank biserial correlation CI95[.96, ∞] |
| Approach intentions | *H*(2) = 171.60, *p* < .001 | *W* = 11663, *p <* .001, rank biserial correlation CI95[.17, ∞] | **1** (p>c) *W* = 15100, *p <* .001, rank biserial correlation CI95[.64, ∞]  **2** (i>c) *W* = 16489, *p <* .001, rank biserial correlation CI95[.80, ∞] |

*Note.* 1 (p>c): comparison: values expected to be higher in person-first language than in the control condition; 2 (i>c): comparison: values expected to be higher in identity-first language than in the control condition

To investigate Hypothesis 2, that is, that the effect of person-first language is stronger for social groups about which individuals hold more important (negative) attitudes, we had planned to verify that participants did, indeed, hold largely negative views on the topics of violent crime and physical disability and that the former topic was considered more important. Information on attitude valence and importance was collected in the pre-test; these analyses are, therefore, based on *N* = 725 participants. The Shapiro-Wilk test indicated that assumptions of normal distribution of the outcome variables ‘valence violent crime’ (*W* = .56, *p* < .001), ‘valence physical disability’ (*W* = .91, *p* < .001), ‘importance violent crime’ (*W* = .89, *p* < .001), and ‘importance physical disability’ (*W* = .91, *p* < .001) were not fulfilled. Hence, we conducted one-tailed Wilcoxon signed-rank tests.

As postulated, attitudes about violent crime were strongly negative ((i.e., testing whether the variables’ mean score was below 3) *M* = 1.50, *SD* = 1.00; *W* = 13023, *p* < .001, rank biserial correlation CI95[-∞, -.88]). The same cannot be said about attitudes towards physical disability (*M* = 4.22, *SD* = 1.37; *W* = 193149, *p* = 1.00, rank biserial correlation CI95[-∞, .81]). An exploratory one-sided Wilcoxon signed-rank test with the reverse alternative hypothesis highlighted that, instead, attitudes towards physical disability were highly positive (*W* = 193149, *p* < .001, rank biserial correlation CI95[.76, ∞]). Indeed, participants rated the topic of physical disability significantly more positive than that of violent crime (*W* = 1840.5, *p* < .001, rank biserial correlation CI95[-∞, -.98])

Next, we aimed to test whether attitudes towards violent crime were more important than those towards physical disability. The Wilcoxon signed-rank test demonstrated that in line with our expectation attitudes towards violent crime (*M* = 3.64, *SD* = 1.12) were considered more important than those towards physical disability (*M* = 3.27, *SD* = 1.15; *W* = 68249, *p* < .001, rank biserial correlation CI95[.32,∞]).

We had pre-registered two-sided equivalence tests for each outcome variable to assess whether the effect of person-first language was stronger for the descriptor pairing “violent criminals/people who have committed a violent crime”. However, in this sub-sample, statistically significant influences of person-first language were confirmed for three outcomes (i.e., ‘negative stereotypes’, ‘negative affect’, ‘approach intentions’), while no significant differences in mean scores on the same measures were identified in the sub-sample that reported views on either the physically disabled or people with a physical disability. Crucially, as physical disability was viewed as strongly positive, the two social groups that were presented in the descriptor pairing differed in terms of valence *and* attitude importance. Thus, Hypothesis 2, which sought to examine the moderating role of only attitude importance, could not be tested as intended.

**Exploratory Analysis**

An alternative analytical approach was therefore chosen to provide nonetheless insights about the moderating role of attitude importance on the effects of person-first language. Namely, interaction effects between attitude importance as reported in the pre-test and exposure to identity-first or person-first language were assessed for all five outcome variables, separately for each descriptor pairing. Results (Table S2.4) showed that for the descriptor pairing “violent criminals/people who have committed a violent crime” there was no significant interaction effect (positive stereotypes: *F*(3, 261) = 1.05, *p =* .369, *R^2^* = .01; negative stereotypes: *F*(3, 261) = 6.56, *p <* .001, *R^2^* = .06; dehumanization: *F*(3, 261) = .93, *p =* .428, *R^2^* = .01; negative affect: *F*(3, 261) = 4.91, *p =* .002, *R^2^* = .05; approach intentions: *F*(3, 261) = 6.45, *p <* .001, *R^2^* = .07).

The same pattern was confirmed for the descriptor pairing “the physically disabled/people with a physical disability” and the outcomes positive stereotypes (*F*(3, 264) = 10.84, *p <* .001, *R^2^* = .11), negative stereotypes (*F*(3, 264) = 5.04, *p =* .002, *R^2^* = .05), dehumanization (*F*(3, 264) = 4.46, *p =* .004, *R^2^* = .05), and approach intentions (*F*(3, 264) = 6.78, *p <* .001, *R^2^* = .07). However, for the outcome negative affect (*F*(3, 264) = 5.53, *p =* .058, *R^2^* = .03), the interaction term was statistically significant, indicating that for participants who were exposed to person-first terminology, stronger importance of the issue physical disability predicted stronger negative affect. Additionally, for the descriptor pairing “the physically disabled/people with a physical disability”, attitude importance was associated with all outcomes except positive stereotypes, suggesting that stronger importance of the issue of physical disability was associated with less negative outgroup perceptions, less negative affect, and stronger approach intentions.

*Table S2.4*

Assessing interaction effects of attitude importance to explore Hypothesis 2

| **Descriptor pairing “violent criminals/people who have committed a violent crime”** | | | | | |  |
| --- | --- | --- | --- | --- | --- | --- |
| Predictor | Positive stereotypes | Negative stereotypes | Dehumanization | Negative affect | Approach intentions | |
| Experimental condition | *B* = -.04 (.08), *p =* .608 | *B* = -.39 (.09), *p <* .001 | *B* = -.10 (.08), *p =* .200 | *B* = -.28 (.09), *p =* .002 | *B* = -.35 (.08), *p <* .001 | |
| Attitude importance | *B* = -.07 (.05), *p =* .169 | *B* = .03 (.05), *p =* .539 | *B* = .00 (.04), *p =* .942 | *B* = .10 (.05), *p =* .073 | *B* = .05 (.05), *p =* .326 | |
| Interaction term | *B* = .03 (.09), *p =* .729 | *B* = .04 (.09), *p =* .670 | *B* = -.06 (.08), *p =* .429 | *B* = .01 (.09), *p =* .895 | *B* = .03 (.09), *p =* .769 | |
| **Descriptor pairing “the physically disabled/people with a physical disability”** | | | | | | |
| Predictor | Positive stereotypes | Negative stereotypes | Dehumanization | Negative affect | Approach intentions | |
| Experimental condition | *B* = -.25 (.08), *p =* .003 | *B* = .10 (.09), *p =* .270 | *B* = .10 (.06), *p =* .095 | *B* = -.02 (.06), *p =* .734 | *B* = .04 (.07), *p =* .618 | |
| Attitude importance | *B* = .22 (.05), *p <* .001 | *B* = -.16 (.06), *p =* .008 | *B* = -.07 (.04), *p =* .073 | *B* = -.09 (.04), *p =* .007 | *B* = -.19 (.05), *p <* .001 | |
| Interaction term | *B* = -.12 (.09), *p =* .177 | *B* = -.01 (.10), *p =* .931 | *B* = -.05 (.06), *p =* .449 | *B* = .13 (.06), *p =* .030 | *B* = .11 (.08), *p =* .168 | |

*Note*. unstandardized coefficients are presented; standard errors are presented in brackets.

**Comparison with the Analysis Presented in the Main Text**

For the descriptor pairing “violent criminals/people who have committed a violent crime”, the analysis presented in the main text also identified a significant interaction between the experimental conditions and attitude importance regarding the outcome ‘dehumanization’. Patterns of interaction effects were the same in both analyses for the descriptor pairing “the physically disabled/people with a physical disability”. The significant associations between the experimental condition and various outcomes that were observed in the analysis here were not replicated in the main text (i.e., the analysis that applied all pre-registered data exclusion rules).
