# Supplementary material for: Does attitude importance moderate the effects of person-first language? A registered report
Source: PLoS One. 2025 Oct 8;20(10):e0332733. doi: 10.1371/journal.pone.0332733 (PMC12507193; doi:10.1371/journal.pone.0332733)
Supplement: S3 File — (DOCX) [file pone.0332733.s003.docx]

**Supplementary Material S3**

*Table S3.1*

Univariate outliers

| **Variable** | **Median** | **MAD** | **Rejection criterion** | **Number of univariate outliers** |
| --- | --- | --- | --- | --- |
| Valence violent crime | 1 | 1.4826 | 1-4.4478 < xi < 1+4.4478 | 7 |
| Valence physical disability | 4 | 5.9304 | 4-17.791 < xi < 4+17.791 | 0 |
| Importance violent crime | 4 | 5.9304 | 4-17.791 < xi < 4+17.791 | 0 |
| Importance physical disability | 3 | 4.4478 | 3-13.343 < xi < 3+13.343 | 0 |
| Positive stereotypes | 3 | 4.4478 | 3-13.343 < xi < 3+13.343 | 0 |
| Negative stereotypes | 3 | 4.4478 | 3-13.343 < xi < 3+13.343 | 0 |
| Dehumanization | 2.75 | 4.077 | 2.75-12.231 < xi < 2.75+12.231 | 0 |
| Negative affect | 2 | 2.965 | 2-8.896 < xi < 2+8.896 | 0 |
| Approach intentions | 3 | 4.4478 | 3-13.343 < xi < 3+13.343 | 0 |

*Note.* MAD = Median * 1.4826; The rejection criterion is defined as Median−3⁎MAD < xi < Median+3⁎MAD
