# Supplementary material for: Does attitude importance moderate the effects of person-first language? A registered report
Source: PLoS One. 2025 Oct 8;20(10):e0332733. doi: 10.1371/journal.pone.0332733 (PMC12507193; doi:10.1371/journal.pone.0332733)
Supplement: S4 File — (DOCX) [file pone.0332733.s004.docx]

**Supplementary Material S4**

Bi-variate correlations of dependent variables in each experimental and the control condition.

*Table S4.1*

Control condition

|  | **Variable** | **1** | **2** | **3** | **4** | **5** |
| --- | --- | --- | --- | --- | --- | --- |
| 1 | Positive stereotypes | 1 |  |  |  |  |
| 2 | Negative stereotypes | -.32 | 1 |  |  |  |
| 3 | Dehumanization | -.66 | .34 | 1 |  |  |
| 4 | Negative affect | -.35 | .56 | .43 | 1 |  |
| 5 | Approach intentions | -.45 | .50 | .55 | .56 | 1 |

*Note.* All correlations are statistically significant at p < .001

*Table S4.2*

“Violent criminals“

|  | **Variable** | **1** | **2** | **3** | **4** | **5** |
| --- | --- | --- | --- | --- | --- | --- |
| 1 | Positive stereotypes | 1 |  |  |  |  |
| 2 | Negative stereotypes | -.42 | 1 |  |  |  |
| 3 | Dehumanization | -.54 | .48 | 1 |  |  |
| 4 | Negative affect | -.37 | .49 | .40 | 1 |  |
| 5 | Approach intentions | -.23  *p* = .010 | .42 | .28  *p* = .002 | .57 | 1 |

*Note.* Unless stated otherwise, all correlations are statistically significant at *p* < .001

*Table S4.3*

“People who have committed a violent crime“

|  | **Variable** | **1** | **2** | **3** | **4** | **5** |
| --- | --- | --- | --- | --- | --- | --- |
| 1 | Positive stereotypes | 1 |  |  |  |  |
| 2 | Negative stereotypes | -.45 | 1 |  |  |  |
| 3 | Dehumanization | -.60 | .51 | 1 |  |  |
| 4 | Negative affect | -.36  *p* = .002 | .60 | .55 | 1 |  |
| 5 | Approach intentions | -.42 | .46 | .49 | .76 | 1 |

*Note.* All correlations are statistically significant at *p* < .001

*Table S4.4*

“The physically disabled“

|  | **Variable** | **1** | **2** | **3** | **4** | **5** |
| --- | --- | --- | --- | --- | --- | --- |
| 1 | Positive stereotypes | 1 |  |  |  |  |
| 2 | Negative stereotypes | -.30 | 1 |  |  |  |
| 3 | Dehumanization | -.63 | .48 | 1 |  |  |
| 4 | Negative affect | -.22  *p* = .045 | .46 | .36 | 1 |  |
| 5 | Approach intentions | -.52 | .46 | .56 | .56 | 1 |

*Note.* Unless stated otherwise, all correlations are statistically significant at *p* < .001

*Table S4.5*

“People with a physical disability“

|  | **Variable** | **1** | **2** | **3** | **4** | **5** |
| --- | --- | --- | --- | --- | --- | --- |
| 1 | Positive stereotypes | 1 |  |  |  |  |
| 2 | Negative stereotypes | -.27  *p* = .004 | 1 |  |  |  |
| 3 | Dehumanization | -.52 | .51 | 1 |  |  |
| 4 | Negative affect | -.10  *p* = .314 | .35 | .24  *p* = .012 | 1 |  |
| 5 | Approach intentions | -.27  *p* = .005 | .30 | .32 | .50 | 1 |

*Note.* Unless stated otherwise, all correlations are statistically significant at *p* < .001
